# NAD^+^ Homeostasis in Diabetic Kidney Disease

**DOI:** 10.3389/fmed.2021.703076

**Published:** 2021-07-21

**Authors:** Jing Xu, Munehiro Kitada, Daisuke Koya

**Affiliations:** ^1^Department of Diabetology and Endocrinology, Kanazawa Medical University, Uchinada, Japan; ^2^Department of Endocrinology and Metabolism, The Affiliated Hospital of Guizhou Medical University, Guiyang, China; ^3^Division of Anticipatory Molecular Food Science and Technology, Medical Research Institute, Kanazawa Medical University, Uchinada, Japan

**Keywords:** nicotinamide adenine dinucleotide, diabetic kidney disease, oxidative stress, sirtuins, PARPs, CD38

## Abstract

The redox reaction and energy metabolism status in mitochondria is involved in the pathogenesis of metabolic related disorder in kidney including diabetic kidney disease (DKD). Nicotinamide adenine dinucleotide (NAD^+^) is a cofactor for redox reactions and energy metabolism in mitochondria. NAD^+^ can be synthesized from four precursors through three pathways. The accumulation of NAD^+^ may ameliorate oxidative stress, inflammation and improve mitochondrial biosynthesis via supplementation of precursors and intermediates of NAD^+^ and activation of sirtuins activity. Conversely, the depletion of NAD^+^ via NAD^+^ consuming enzymes including Poly (ADP-ribose) polymerases (PARPs), cADPR synthases may contribute to oxidative stress, inflammation, impaired mitochondrial biosynthesis, which leads to the pathogenesis of DKD. Therefore, homeostasis of NAD^+^ may be a potential target for the prevention and treatment of kidney diseases including DKD. In this review, we focus on the regulation of the metabolic balance of NAD^+^ on the pathogenesis of kidney diseases, especially DKD, highlight benefits of the potential interventions targeting NAD^+^-boosting in the treatment of these diseases.

## Introduction

The kidney is rich in mitochondria where generates the majority of adenosine triphosphate (ATP) required for various cellular metabolic activities (1). Nicotinamide adenine dinucleotide (NAD^+^) is considered as a critical cofactor and intermediary for turning fuel into energy through redox reaction (2). During the reduction reactions, which participate in glycolysis, fatty acid oxidation, and tricarboxylic acid (TCA) cycle, NAD^+^ serves as a hydrogen ion acceptor to generate its reduced form, NADH. Correspondingly, NADH participates in mitochondrial ATP generation by removing hydrogen ions through oxidation reactions (2, 3). NAD^+^ is also a cosubstrate for enzymes involved in non-redox reactions such as the sirtuins family, poly (ADP-ribose) polymerases (PARPs), and the cyclic ADP-ribose (cADPR) synthases, such as CD38 and CD157, to participate in the regulation of multiple cellular process, especially in energy metabolism (4, 5). Decreased cellular NAD^+^ concentrations and NAD^+^ /NADH ratio are closely related to the pathogenesis of multiple age-related and metabolic diseases, including diabetic kidney disease (DKD) (6). Thus, boosting NAD^+^ levels may be a potential therapeutic strategy for preventing the pathogenesis of DKD. In this review, we present the biogenesis of NAD^+^ including its synthesis, degradation, and other regulatory signaling particularly mitochondrial quality control in the development and progression of DKD, highlight the role of NAD^+^ supplementation and potential therapy targeting increasing NAD^+^ on the treatment of DKD.

## Biology of NAD^+^

### NAD^+^ Anabolism

**Figure 1:**
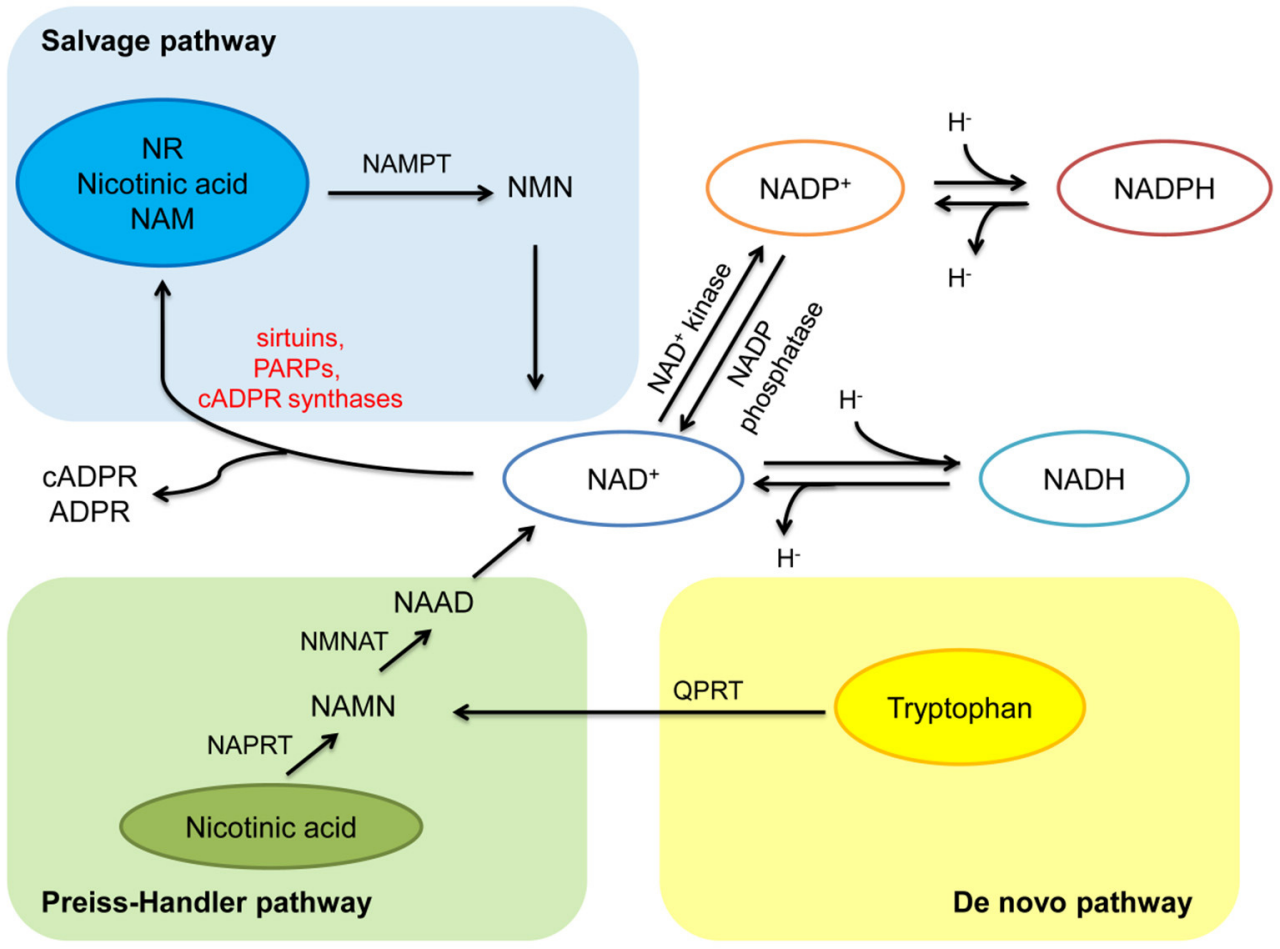
Anabolism and catabolism of NAD^+^. NAD^+^ is synthesized from four precursors including nicotinamide (NAM), tryptophan, nicotinic acid, and nicotinamide riboside (NR) through three pathways (salvage pathway, *de novo* pathway, and Preiss-Handler pathway). In salvage pathway, the precursors are converted into an intermediate called nicotinamide mononucleotide (NMN) through nicotinamide phosphoribosyltransferase (NAMPT). Then NMN is converted into NAD^+^ via nicotinamide mononucleotide adenylyltransferase (NMNAT). NAD^+^ generated by this pathway is consumed by multiple enzymes including sirtuins, PARPs, and cADPR synthases, to generate NAM, and reused in salvage pathway. In Preiss-Handler pathway, nicotinic acid is converted into nicotinate mononucleotide (NAMN) through nicotinic acid phosphoribosyltransferase (NAPRT), then NAMN is converted into nicotinic acid adenine dinucleotide (NAAD) through NMN transferase (NMNAT), further generate NAD^+^. In *de novo* pathway, tryptophan is converted into NAMN via quinolinate phosphoribosyltransferase (QPRT), and then into NAD^+^ via the Preiss-Handler pathway. In redox reactions, NAD^+^ can be phosphorylated to NADP^+^ by NAD^+^ kinase. NADP^+^ can be dephosphorylated to NAD^+^ by NADP phosphatase. Both the oxidized forms (NAD^+^ and NADP^+^) serve as hydride acceptors to generate their reduced forms (NADH and NADPH).

NAD^+^ can be synthesized from four precursors including nicotinamide (NAM), tryptophan, nicotinic acid (a form of niacin, also known as vitamin B3), and nicotinamide riboside (NR), obtained from daily diet (including milk, meats, nuts, et al.), through three pathways (2, 5) (Figure 1). In mammals, the main synthetic pathway for NAD^+^ is salvage pathway (7, 8). In salvage pathway, the precursors including NAM, nicotinic acid and NR are converted into an intermediate called nicotinamide mononucleotide (NMN) through a rate-limiting enzyme, nicotinamide phosphoribosyltransferase (NAMPT). The intermediate NMN can be converted into NAD^+^ via nicotinamide mononucleotide adenylyltransferase (NMNAT). NAD^+^ generated by this pathway is consumed by multiple enzymes including sirtuins, PARPs, cADPR synthases, to generate NAM, which can be reused in salvage pathway again (2, 5). In Preiss-Handler pathway, nicotinic acid obtained from the daily diet can be converted into nicotinate mononucleotide (NAMN) and nicotinic acid adenine dinucleotide (NAAD) via some key enzymes such as nicotinic acid phosphoribosyltransferase (NAPRT) and NMN transferase (NMNAT), respectively, then further generate NAD^+^ (5). In kidney, except salvage pathway, *de novo* pathway is another main source of NAD^+^ (9). In *de novo* pathway, tryptophan can be converted into NAMN via quinolinate phosphoribosyltransferase (QPRT), and then into NAD^+^ via the Preiss-Handler pathway (5). The regulation of rate-limiting enzymes in these pathways and the supplementation of precursors and intermediates may be potential treatments for metabolic related disorder including diabetes and obesity (7, 10, 11).

### NAD^+^ Catabolism

The catabolism of NAD^+^ mainly involves three types of enzymes, including sirtuins, PARPs, and cADPR synthases (5) (Figure 1). These NAD^+^-degrading enzymes are involved in a variety of metabolic pathways and play key roles in the pathogenesis of aging-related diseases.

Sirtuins (SIRT1-7), a highly conserved homologous family from bacteria to mammals, is recognized as antiaging molecules (12). Among them, SIRT1, 6, 7 are mainly expressed in the nucleus, SIRT2 is mainly expressed in the cytoplasm, and SIRT3, 4, 5 are highly expressed in mitochondria (13). Deacetylation dependent on the consuming of NAD^+^ is the most important function of sirtuins, which involved in multiple metabolic regulation (14). Both calorie restriction (CR) (15, 16) and AMP-activated kinase (AMPK) (4) can induce SIRT1 activity by sensing elevated NAD^+^ concentration. SIRT1 deacetylates histones, including H4, H3, and H1 (17) and other transcription factors to regulate the posttranslational modifications of target genes and the expression of downstream proteins. SIRT1 deacetylates nuclear factor-κB (NF-κB) to suppress inflammatory signaling pathway (18), deacetylates signal transducer and activator of transcription 3 (STAT3) to protect from apoptosis (19), deacetylates microtubule-associated protein 1A/1B-light chain 3 (LC3) to activate autophagy (20), and deacetylates peroxisome proliferator-activated receptor gamma coactivator 1α (PGC-1α) (21, 22), Mfn (mitofusin)1 and 2 (23, 24), further participate in the regulation of mitochondrial biogenesis, oxidative stress and fusion process, respectively. SIRT2 deacetylates the forkhead box O (FOXO) 3a to suppress oxidative stress (25), deacetylates PGC-1α to regulate fatty acid oxidation (26). In erythrocytes, in response to increased oxidative stress, SIRT2 deacetylates glucose-6-phosphate dehydrogenase (G6PD), a key enzyme involved in pentose phosphate pathway, to increase the production of nicotinamide adenine dinucleotide phosphate (NADPH) (27). SIRT3 can also be activated by CR and AMPK through increased NAD^+^ levels (28, 29). SIRT3 deacetylates superoxide dismutase 2 (SOD2) and isocitrate dehydrogenase 2 (IDH2) to alleviate oxidative stress (30), deacetylates optic atrophy 1 (OPA1) to regulate mitochondrial fusion (31), deacetylates PGC-1α to improve mitochondrial biogenesis (32). Different from SIRT1-3, which predominately function as deacetylases, SIRT4 functions as ADP-ribose via NAD^+^ utilization and is responsible for cellular insulin secretion (33). SIRT4 suppresses insulin secretion via inhibiting glutamate dehydrogenase (GDH) activity through ADP-ribosylation (33) and SIRT4 knockdown increases fatty acid oxidation in liver and muscle cells (34). Although SIRT5 is also a NAD^+^-dependent deacetylase (35), it predominantly presents desuccinylated effect on IDH2, which suppresses cellular oxidative stress (36). SIRT6 deacetylates histone H3K9 and H3K56 to restore high glucose-impaired mitochondrial dysfunction, suppresses apoptosis and inflammation in potocytes (37). SIRT7 is less studied, previous study showed it suppresses the nuclear export of NF-κB p65 via deacetylating Ras-related nuclear antigen in the nucleus (38). These evidences combine sirtuins with mitochondrial biosynthesis, oxidative stress, apoptosis, and inflammation, suggesting a crucial role of cellular NAD^+^ levels regulated by sirtuins in maintaining cellular homeostasis.

PARP is a family containing at least 17 enzymes. Among them, PARP-1 is the most the most widely studied enzyme which can catalyze the synthesis of ADP-ribose on target proteins in response to DNA damage and genotoxic stress via NAD^+^ consumption (39, 40). High glucose induced DNA damage may contribute to excessive activation of PARP (41). Subsequent studies have also confirmed that PARP-1 is closely related to the activation of oxidative stress and inflammation (42). Selective PARP-1 inhibitor suppresses oxidative stress, inflammation via activating SIRT1/ PGC-1α signaling in diabetic mice (43) (**Figure 3A** and Table 1).

**Table 1:** Deacetylated targets of NAD^+^-consuming enzymes in DKD.

**Enzymes**	**Targets**	**Effects**	**Interventions**
SIRT1	PGC-1α p53 NF-κB p65 STAT3 LC3 Smad4 FOXO3a	Mitochondrial biogenesis↑ Apoptosis↓ Inflammation↓ Inflammation↓ Autophagy↑ fibrosis↓ Oxidative stress↓ Apoptosis↓	Dietary restriction (18) Resveratrol (44–46) BF175 (47) SRT1720 (48) SRT2379 (49) Metformin (50, 51) Canagliflozin (52)
SIRT2	FOX3a	Oxidative stress↓	Caloric restriction (25)
SIRT3	SOD2 IDH2 p53	Oxidative stress↓ Oxidative stress↓ Apoptosis↓ Fibrosis↓	Caloric restriction (28, 53) AICAR (32) Empagliflozin (54)
SIRT6	H3K9 H3K56	Apoptosis↓ Inflammation↓ Mitochondrial function↑	-
PARPs	PARPs inhibition	Oxidative stress↓ Apoptosis↓ Inflammation↓	Tempol (55) INO-1001 (56) PJ-34 (55–57) 3-aminobenzamide (58, 59)
CD38	CD38 inhibition	Oxidative stress↓ Inflammation↓	Apigenin (30, 60–62) 78c (63) Quercetin (64)

The cADPR synthases CD38 and its homolog CD157 can be induced by inflammatory cytokines, which is associated with aging-related decrease of NAD^+^ levels (65). CD38 catalytic activity via degrading NAD^+^ mainly generates NAM and cADPR through salvage pathway. One molecule of cADPR generation via CD38 for every 100 molecules of NAD^+^ hydrolyzed (66). CD38 also degrades NAD^+^ precursor NR and intermediate NMN (67). CD38 and CD157 also serve as Ca^2+^-transporting second messengers to stimulate Ca^2+^ release, which participates in the regulation of cardio muscle, renal vasoconstriction (68, 69), and insulin secretion in pancreatic β cells (70). CD38 inhibition in pancreatic β cells suppresses insulin signaling (71, 72). CD38 knockout mice show decreased cADPR and increased NAD^+^ concentrations, which may protect from inflammation, apoptosis, oxidative stress, and high-fat diet induced obesity (30, 73, 74) (**Figure 3B** and Table 1).

### NADP^+^ and NADPH

**Figure 2:**
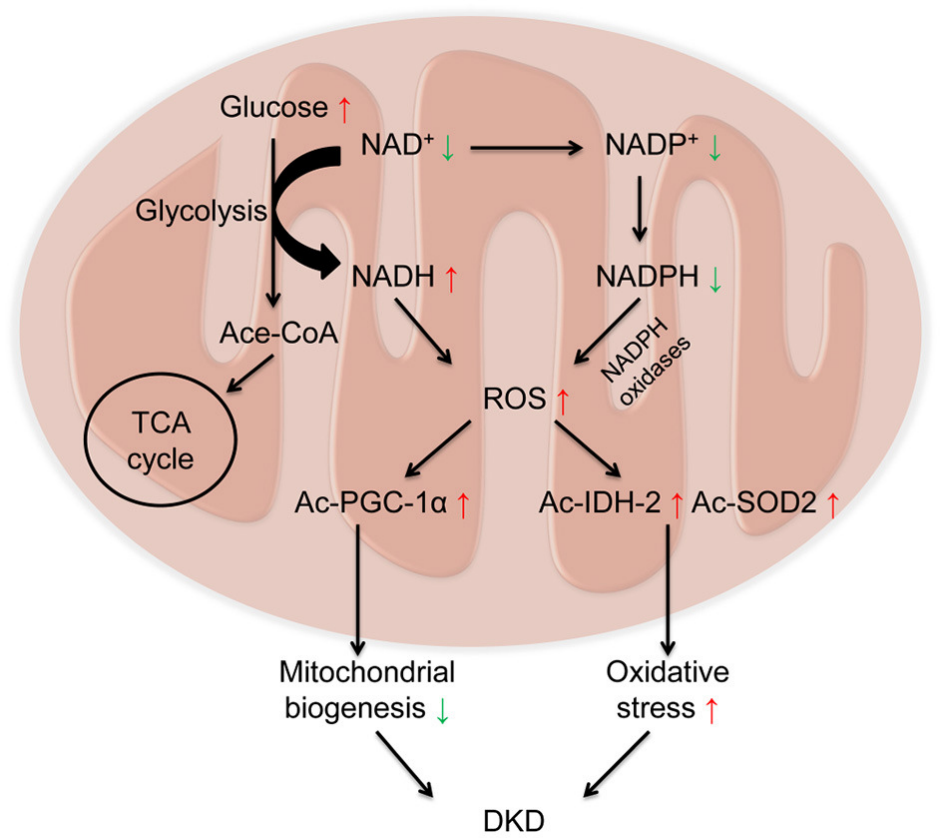
Decreased NAD^+^ and increased Reactive oxygen species (ROS) in diabetic kidney disease (DKD). Under diabetic condition, glucose is metabolized to acetylated-CoA, which enters the tricarboxylic acid cycle (TCA) cycle via consuming NAD^+^. On one hand, NAD^+^ receives hydrogen to be reduced into NADH, leading to the overload of NADH and the elevated levels of ROS. On the other hand, NADPH oxidases transfer electrons from NADPH and interact with oxygen to form superoxide to aggravate the production of ROS. Overloaded ROS in mitochondria results in the acetylation of peroxisome proliferator-activated receptor gamma coactivator 1α (PGC-1α) to impair mitochondrial biogenesis, and acetylation of isocitrate dehydrogenase 2 (IDH2) and superoxide dismutase 2 (SOD2) to aggravate oxidative stress, ultimately leading to the progression of DKD.

In redox reactions, NAD^+^ can also be phosphorylated to NADP^+^ by NAD^+^ kinase. In contrast, NADP^+^ can be dephosphorylated to NAD^+^ by NADP phosphatase. Both The oxidized forms (NAD^+^ and NADP^+^) serve as hydride acceptors to generate their reduced forms (NADH and NADPH) (5, 75). These processes involve in the synthesis and consumption of NAD^+^ form a redox reaction cycle (Figure 1). Mitochondria are the main organelle that produces ROS in kidney. NADPH has antioxidant effects in mitochondrial biogenesis, while NADPH oxidases transfer electrons from NADPH and interact with oxygen to form superoxide to aggravate the production of ROS in mitochondria, which is also a main source of ROS in kidney (76) (Figure 2).

## Regulation of NAD^+^ Levels in the Pathogenesis of DKD

The pathogenesis of DKD involves multiple mechanisms, including mitochondrial dysfunction, oxidative stress, and inflammation (77). Imbalance of NAD^+^ and NADH is a hallmark of diabetes and its chronic complications (78). Under diabetic condition, with the activation of glycolytic pathway, glucose is metabolized into acetylated-CoA, which enters the TCA cycle. During this procedure, NAD^+^ obtain hydrogen to become NADH via the catalysis of glyceraldehyde 3-phosphate dehydrogenase and pyruvate dehydrogenase, leading to the overload of NADH and the elevated production of reactive oxygen species (ROS), which further results in oxidative stress (79, 80). The decline in NAD^+^ levels also results in the acetylation of many proteins involved in oxidative stress and mitochondrial biogenesis, such as IDH2, SOD2(30), and PGC-1α (44), which ultimately leads to the progression of DKD (Figure 2). Therefore, as a key organelle for cellular redox reactions, mitochondrial function is essential for the maintenance of NAD^+^ levels. In this part, we mainly focus on the effects of sirtuins (especially SIRT1-6), PARPs, and cADPR synthases (particularly CD38) in the pathogenesis of DKD (Table 1).

### Nuclear Sirtuins

SIRT1 deacetylates a variety of proteins involved in mitochondrial biogenesis, oxidative stress, inflammation apoptosis and autophagy via utilizing cellular NAD^+^ (81, 82). CR is a crucial activator of SIRT1 activity via increasing intracellular NAD^+^ levels. AMPK also enhances SIRT1 activity by increasing NAD^+^ to regulate the deacetylation of SIRT1 targets proteins (4, 83). In turn, SIRT1 can deacetylate liver kinase B1 (LKB1), a classic AMPK activating kinase to regulate the activity of AMPK (84). SIRT1 have confirmed its beneficial effects via NAD^+^ dependent deacetylation on amelioration of mitochondrial biogenesis, suppression of oxidative stress, fibrosis, inflammation, and apoptosis in kidney (82) (Table 1). Although mainly expressed in the nucleus, SIRT1 has been implicated in mitochondrial functions. SIRT1 deacetylates PGC-1α to increase mitochondrial biogenesis and mitochondrial fatty acid oxidation (21, 22) and attenuate high glucose-induced mitochondrial oxidative stress in potocytes (44, 45). Moreover, SIRT1 is related to autophagy and mitochondrial autophagy (mitophagy). Previous studies demonstrated that SIRT1 knockout mouse embryonic fibroblasts could not activate autophagy under starvation (85) and SIRT1 deacetylates nuclear LC3 to initiate autophagy (20). Increasing SIRT1 activity induced by elevated NAD^+^/NADH ratio results in increased mitochondrial membrane potential, LC3-II and proteins that regulate mitochondrial fusion and fission (86). SIRT1 reduced FOXO3a acetylation, preventing podocytes from oxidative stress and apoptosis (87). With the depletion of cellular NAD^+^ level in diabetic kidney, the activity of SIRT1 was suppressed, leading to acetylation of p53 and consequently cell death via apoptosis (82, 88). Our previous study and other research also demonstrated diabetic animal models showed decreased SIRT1 levels and increased acetylation of NF-κB p65 and STAT3, accompanying with elevated inflammation-related genes, leading to the injury and apoptosis of potocytes and proximal tubule (18, 19). SIRT1 also suppresses advanced glycation end-product (AGE)-induced diabetic renal fibrosis through antioxidative effects (89) and reduces epithelial-to-mesenchymal transition (EMT) to ameliorate injury-induced kidney fibrosis via deacetylating mothers against decapentaplegic homolog 4 (Smad4) in tubular epithelial cells (90). Besides, SIRT1 alleviates kidney fibrosis via suppresses transforming growth factor β (TGF-β) pathway in mesangial cells, proximal tubular cells and endothelial cells (89, 91–93).

The NAD^+^-dependent deacetylated effects of SIRT6 exert renoprotective effects in diabetic rodent models and high-glucose treated cells (Table 1). Previous studies reported that the expression of SIRT6 is decreased under diabetic condition (94, 95). SIRT6 inhibits high glucose-induced mitochondrial dysfunction and apoptosis in potocytes via deacetylating histone H3K9 and H3K56 (95). Proximal tubule-specific SIRT6 knockout mice exhibit enhanced fibrogenic extracellular matrix remodeling in kidney under high glucose condition (96). Podocyte-specific knockout SIRT6 mice showed renal injury and proteinuria under diabetic condition. SIRT6 inhibits Notch 1 and 4 via deacetylating histone H3K9, to suppress inflammation, apoptosis in potocytes (37) and overexpression of SIRT6 in macrophages protected podocytes from high glucose-induced renal injury (97).

### Cytoplasmic and Mitochondrial Sirtuins

Studies between SIRT2 and DKD are limited. One research showed that SIRT2 is highly expressed in kidney and can deacetylate FOXO3a to decrease cellular ROS in the kidney of caloric-restricted mice (25) (Table 1).

Mitochondrial SIRT3 is a NAD^+^-dependent deacetylase, which predominantly exerts antioxidant activities on preventing aging-related diseases (98, 99). SIRT3 activity is decreased in diabetic patients and rodent animal models (30, 100, 101). SIRT3 deficiency presents impaired insulin secretion, renal fibrosis, elevated acetylation of mitochondrial proteins and increased mitochondrial oxidative stress (30, 102). Rat glomerular mesangial cells exposed to high glucose showed decreased NAD^+^/NADH ratio and SIRT3 activity, leading to oxidative stress and mesangial hypertrophy (101). Our previous studies observed decreased intracellular NAD^+^/NADH ratio and SIRT3 activity in diabetic rats, resulting in the activation of acetylated-SOD2 and acetylated-IDH2 in kidney mitochondria and tubular cells, then ultimately increasing ROS levels to aggravate oxidative stress (30, 103). Another study indicated overexpression of SIRT3 can ameliorated high glucose induced oxidative stress and apoptosis via Protein kinase B (Akt)/FOXO signaling in human renal tubular epithelial cells (104). In addition, SIRT3 deficiency also has a pathogenic effect on acute kidney injury (AKI). On one hand, SIRT3 deficiency increased dynamin related protein 1 (Drp1) and decreased OPA1 and PGC-1α, leading to a shift of mitochondria from fusion to fission, which exacerbates cisplatin-AKI and stress (31, 32). On the other hand, SIRT3 inhibition acetylated SOD2 and p53, leading to oxidative stress and apoptosis in ischemia/reperfusion -induced AKI (105) (Table 1).

SIRT4 functions as NAD^+^-dependent ADP ribosyltransferase to participate in the regulation of insulin secretion in β cells (106). SIRT4 ADP-ribosylates and suppresses GDH, a key enzyme in glutamine metabolism and ATP production, while suppression of SIRT4 activates glucose-induced insulin secretion (33, 106). SIRT4 knockout mice exert elevated basal and stimulated insulin secretion via GDH activation, resulting in glucose intolerance and insulin resistance (107). The effects of SIRT4 in renal function are limited. SIRT4 overexpression suppresses high glucose- induced overproduction of ROS and inflammatory cytokine including TNFα, IL-6, IL-1β, which protects podocytes from oxidative stress and inflammation (108).

SIRT5 is another mitochondrial NAD^+^-dependent deacetylase, involved in the regulation of mitochondrial quality control. Other post-translational modifications of SIRT5 include demalonylation and desuccinylation to participate in glycolysis, oxidative stress, and fatty acid oxidation (36, 109–112). In mouse liver, SIRT5 expression is suppressed by AMPK and activated by PGC-1α. SIRT5 overexpression in HepG2 cells increased ATP synthesis and oxygen consumption (113). SIRT5 knockout mice showed activated malonylation (114) and glutarylation (115) in multiple organs including kidney. SIRT5 induced the acetylation of NF-κB p65 and its downstream inflammatory cytokines, such as IL-6, TNFα, and monocyte chemoattractant protein 1 (MCP-1) (116). The role of SIRT5 in DKD is limited. However, some studies indicated that SIRT5 protects from AKI via increasing Nrf2 to suppress apoptosis (117) and regulating fatty acid oxidation to improve mitochondrial function in proximal tubule (118).

### PARPs

**Figure 3:**
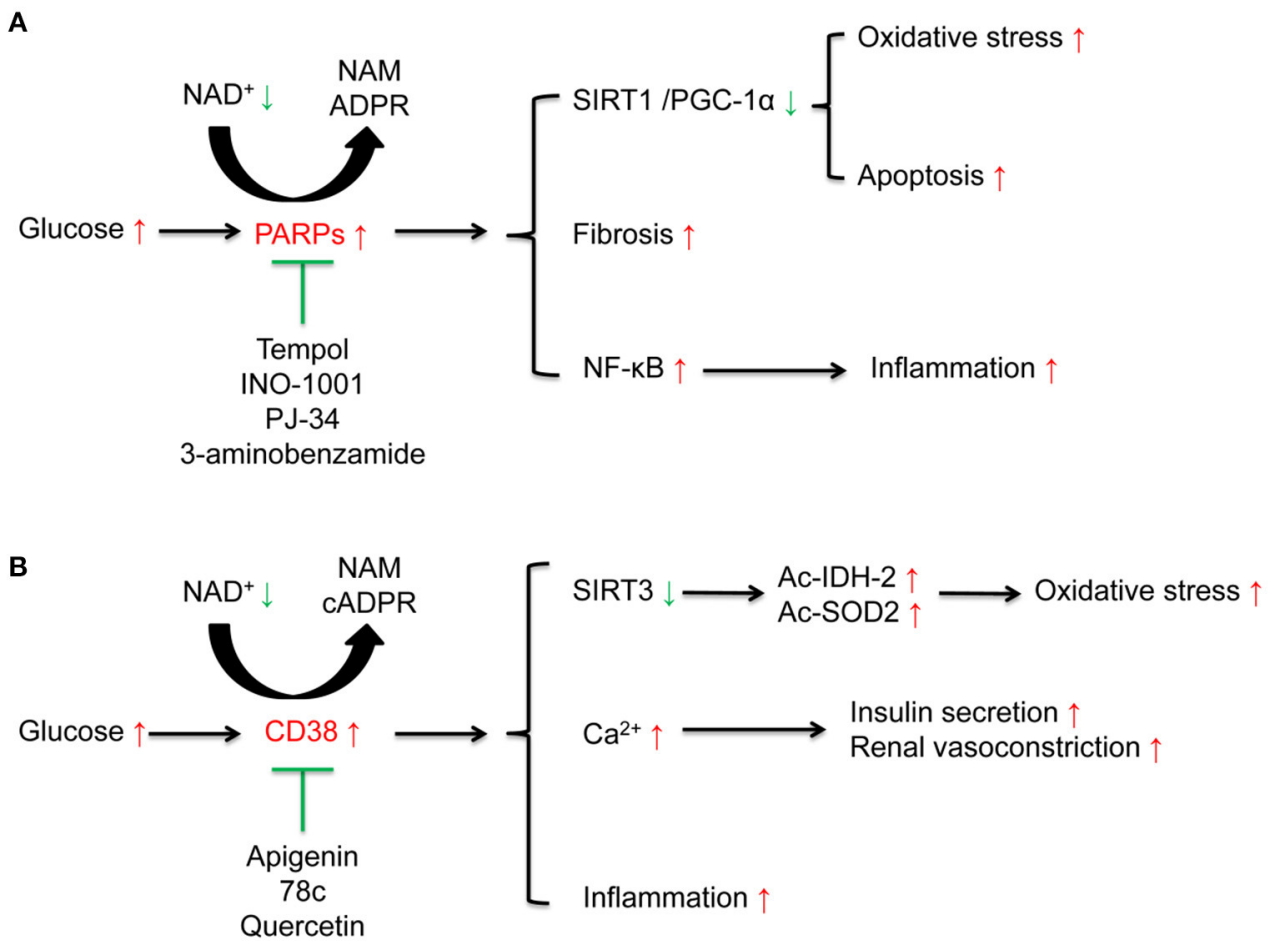
Effects of poly (ADP-ribose) polymerases (PARPs), and the cyclic ADP-ribose (cADPR) synthases CD38 in diabetic kidney disease (DKD). **(A)** High glucose activates NAD^+^ consuming enzyme PARPs. The activated PARPs can induce oxidative stress and apoptosis by inhibiting the sirtuins 1(SIRT1)/peroxisome proliferator-activated receptor gamma coactivator 1α (PGC-1α) signaling, accelerate renal fibrosis, and enhance inflammation by activating nuclear factor-κB (NF-κB). PARP inhibitors such as tempol, INO-1001, PJ-34, 3-aminobenzamide, can ameliorate these alterations induced by high glucose. **(B)** High glucose activates NAD^+^ consuming enzyme CD38. On one hand, CD38 induces the acetylation of isocitrate dehydrogenase 2 (IDH2) and superoxide dismutase 2 (SOD2) to aggravate oxidative stress via suppression of SIRT3 activity and is closely related to inflammation. On the other hand, CD38 is responsible for insulin secretion and renal vasoconstriction via regulating Ca^2+^ release. CD38 inhibitors including apigenin, 78c, and quercetin can suppress these alterations induced by high glucose.

Diabetic rodent models present hyper-activation of PARP and increased consumption of NAD^+^ in renal cortex. The activation of PARP is closely related to elevated levels of endothelin-1 (ET-1), a potent vasoconstrictor, and ET receptors in kidney (58, 59, 119, 120). PRAP activation is also responsible for apoptosis (55, 121, 122), inflammation and fibrosis (123), which leads to the progression of DKD (Figure 3A and Table 1). Moreover, PARP activation exacerbates oxidative stress via consuming NAD^+^ (55, 59). PARP-1 deficiency in diabetic mice ameliorates high glucose-induced kidney hypertrophy, mesangial expansion, collagen deposition, and urinary albumin (120).

### CD38

CD38 contributes to cellular NAD^+^ degradation and is involved in the regulation of cellular glucose metabolism and insulin secretion (124). Compared to the wild type mice, CD38 knockout mice present higher NAD^+^ levels in kidney (73). The functions of CD38 in kidney partially link to nuclear and mitochondrial sirtuins, especially SIRT1 and SIRT3. CD38 inhibition protects from high-fat diet-induced obesity via NAD^+^-dependent SIRT1/PGC-1α signaling (74) and attenuates renal vasoconstriction caused by angiotensin II, ET-1, and norepinephrine (69). Our previous studies demonstrated that high glucose induced-CD38 is responsible for the decreased NAD^+^/NADH ratio and SIRT3 activity inhibition, further results in oxidative stress characterized as elevated acetylated-SOD2 and acetylated-IDH2 in renal tubular cells (30, 103) (Figure 3B and Table 1).

## Potential Interventions Targeting on NAD^+^ in the Treatment of DKD

Based on the effect of maintaining intracellular NAD^+^ stability, interventions targeting the activation of the NAD^+^ synthesis pathway and the inhibition of the metabolic pathway have become potential therapeutic directions.

### Supplementation of NAD^+^ Synthesis

NAD^+^ supplementary therapy can be derived from supplementation of NAD or precursors and intermediates of NAD^+^. Rat glomerular mesangial cells incubated with NAD suppresses high glucose induced mesangial hypertrophy via SIRT1 and SIRT3 mediated-AMPK/mTOR pathway (101). NR supplementation increases NAD^+^ and induces the activity of SIRT1 and SIRT3, which protects from oxidative stress, improves insulin sensitivity (11) and ameliorates hepatic inflammation via suppressing NLRP3 inflammasome in T2DM mice (125). Administration of exogenous NMN, a key intermediate of NAD^+^ synthesis, significantly induces NAD^+^ levels, resulting in the improvement of impaired glucose tolerance, enhancement of insulin sensitivity and suppression of inflammation characterized as decreased acetylated NF-κB p65 in liver of high fat diet- and age-induced diabetic mice (10). NMN treatment also suppresses inflammatory cytokines including TNFα, IL-1β, restores impaired β cell function in islet of fructose-induced diabetic mice. In kidney, NMN treatment alleviates inflammatory and fibrosis in glomerular mesangial cells (126) and STZ-induced diabetic rats via inhibiting endogenous NAMPT (127). A recent study also demonstrated that short-term NMN administration (for 2 weeks) increases NAD^+^ levels, SIRT1 expression and NAD^+^ salvage pathway in kidney, ameliorating urinary albumin excretion, mesangium expansion, and foot process effacement in db/db mice (128). Besides, supplementation of NMN increased NAD^+^ level and protected mice from cisplatin-induced AKI via activating SIRT1 and suppressing the c-Jun N-terminal kinase (JNK) signaling (129).

However, since NAD^+^ synthesis is a complex process involving multiple enzymes, signaling, and metabolites, benefits of supplementation of exogenous NAD^+^ intermediates are still controversial. Previous study showed that NAMPT, a limiting enzyme of NAD^+^ synthesis, is increased in streptozotocin (STZ)-induced diabetic rats, which may be an adaptive, protective response to high glucose-induced inflammation, while exogenous NAMPT may induce inflammation in tubular cells (127, 130). Endogenous NAMPT induces inflammatory and fibrosis in glomerular mesangial cells and STZ-induced diabetic rats through activating NF-κB p65 and suppressing SIRT1 (127). Besides, some studies also showed that exogenous NR administration had no benefits in young healthy animals or humans (131, 132). The results of clinical trials of NAD^+^ supplementation may not reverse AKI. Supplementation of NAM could not relieve inflammation, renal dysfunction and kidney injury in animals and patients of AKI (133). Another randomized, double-blind, placebo-control study demonstrated that although combination of NR supplementation and pterostillbene, a sirtuins activator, can increase NAD^+^ levels after 48 h treatment, there was no benefit on renal function including creatinine and estimated glomerular filtration rate in patients with AKI (134). More researches are needed to identify whether supplementation of certain intermediates in NAD^+^ synthesis can benefit by increasing the intracellular NAD^+^ concentration.

### Activation of Sirtuins

Caloric/dietary restriction is an effective way to activate sirtuins and protects the progression of DKD. Dietary restriction ameliorated kidney inflammation in diabetic mice via activating SIRT1 to inhibit acetylated-NF-κB (18). Calorie restriction reduces renal oxidative stress and inflammation by SIRT2 (25) and attenuates palmitate-induced ROS production and inflammation in proximal tubular cells via SIRT3-mediated deacetylation (28, 53).

**Figure 4:**
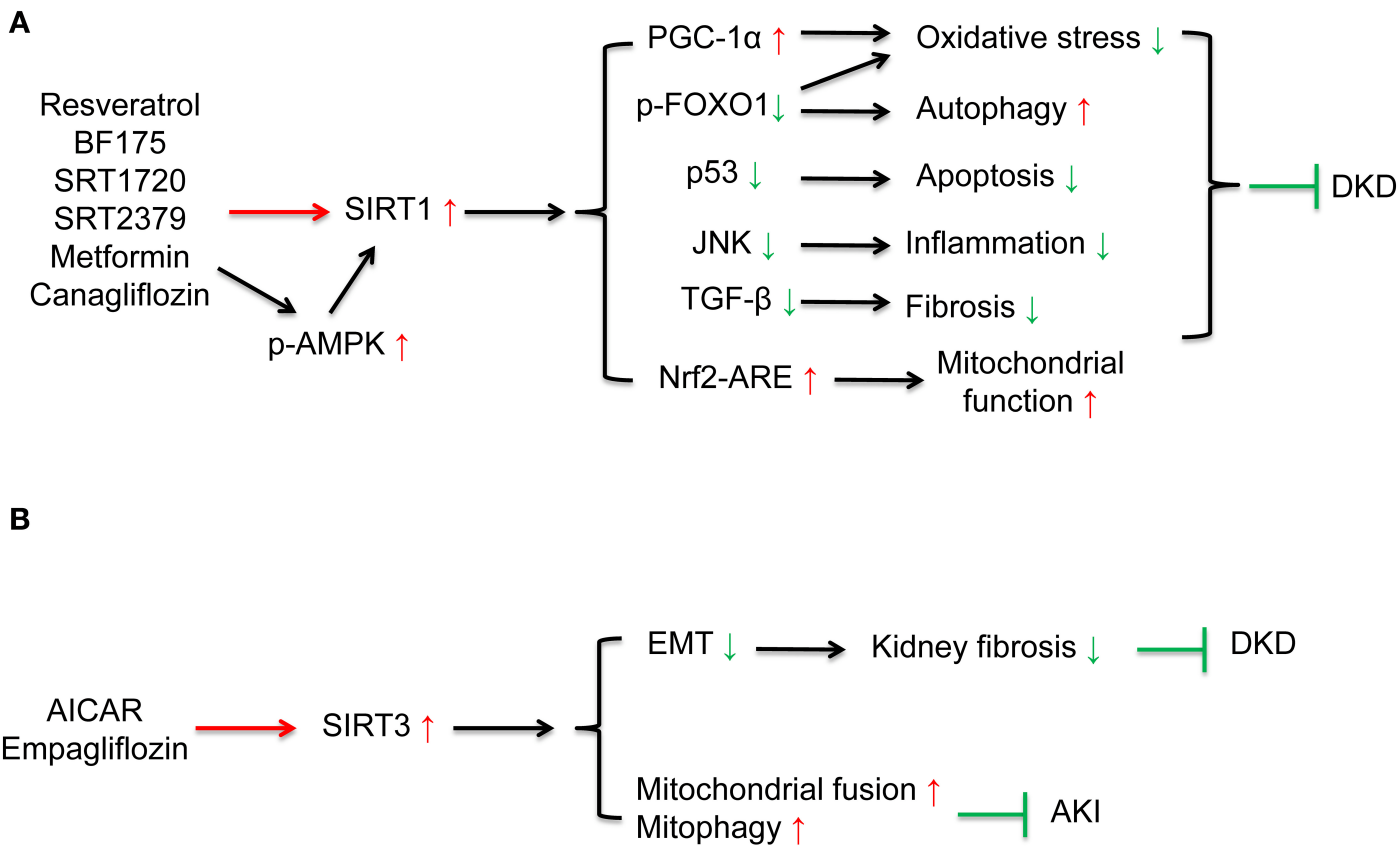
Drug interventions on sirtuins activation in the treatment of kidney diseases. **(A)** SIRT1 activation can ameliorate oxidative stress, apoptosis, fibrosis, inflammation, restore autophagy, and mitochondrial function. SIRT1 agonists, resveratrol, BF175, SRT1720, SRT2379, and some anti-diabetic drugs including metformin and canagliflozin can activate SIRT1 to protect against DKD. **(B)** AICAR can increase mitochondrial fusion and mitophagy via activating SIRT3 to protect against DKD. SGLT2 inhibitor empagliflozin can ameliorate kidney fibrosis via restoring high glucose-suppressed SIRT3 to protect against DKD.

Multiple sirtuin activating compounds have been identified to protect from the progression of DKD via suppression mitochondrial oxidative stress, apoptosis, and inflammation (12). SIRT1 agonists, resveratrol (44–46), BF175 (47), ameliorated mitochondrial oxidative stress and apoptosis in podocytes of diabetic mice via regulating SIRT1/PGC-1α and SIRT1/p53 signaling. Resveratrol also ameliorated high glucose-induced mitochondrial dysfunction via activating SIRT1/Nrf-antioxidant response element (ARE) pathway (135). SRT1720 attenuated renal fibrosis and oxidative stress (48). SRT2379 inhibited LPS-stimulated JNK and IκB kinase (IKK) inflammatory pathways in macrophages (49). AMPK agonist, AICAR reduced cisplatin-induced AKI and improved renal function via the activating SIRT3 deacetylation effect and further activating mitochondrial fusion process and mitopahgy (32). In addition to these compounds under development, some anti-diabetic drugs which have been widely used clinically can also activate sirtuins. Metformin, the first-line medication for T2DM treatment, is an AMPK and sirtuin agonist. Previous studies have confirmed that metformin reduced oxidative stress, enhanced autophagy, and ameliorated insulin resistance by activating AMPK/ SIRT1 /FOXO1 signaling in rat mesangial cells and potocytes, further protected against the pathogenesis of DKD (50, 51). SGLT2 inhibitors are effective anti-diabetic drugs which present renoprotective effects. Previous studies showed SGLT2 inhibitors canagliflozin reversed high glucose-induced SIRT1 suppression (52, 136) in human renal tubular cells and db/db mice to protect against DKD. Our research indicated that SGLT2 inhibitor empagliflozin restored high glucose-suppressed SIRT3, which in part suppressed EMT and kidney fibrosis (54) (Figure 4 and Table 1).

### Inhibition of PARPs

Given the role of PARPs in high glucose-induced oxidative stress, apoptosis and inflammation, inhibitors targeting PARP may be a potential targets for the treatment of DKD. Tempol reduces podocytes apoptosis via suppressing PARP signaling in STZ-induced diabetic rats (55). PARP inhibitors, INO-1001 and PJ-34, suppressed high glucose induced ROS levels and nuclear NF-κB in potocytes and db/db mice and potocytes apoptosis in STZ-induced diabetic rats (55, 56). Another PARP inhibitor, 3-aminobenzamide, inhibited high glucose induced oxidative stress and ET-1 expression in diabetic rodent models (58, 59). The effect of PARP inhibitor is also related to SIRT1 activation. One study demonstrated that PARP inhibitor PJ-34 interacted with SIRT1 to suppress the accumulation of renal extracellular matrix via activating AMPK/ PGC-1α signaling in db/db mice (57) (Figure 3A and Table 1).

### Inhibition of CD38

Suppression NAD^+^-degrading enzyme such as CD38 is an effective way to increasing endogenous NAD^+^ level and restores impaired mitochondrial functions (63, 67). CD38 inhibitor, apigenin, increased NAD^+^ to decrease acetylation of p53 and NF-κB p65 (60). Based on these studies, our research demonstrated that apigenin suppressed high glucose-induced acetylation of SOD2 and IDH2 to ameliorate mitochondrial oxidative stress, via increasing NAD^+^/NADH ratio and SIRT3 activity in renal tubular cells of diabetic rats (30). Apigenin also ameliorated renal inflammation via inhibiting mitogen-activated protein kinase (MAPK) pathway in STZ-induced diabetic rats (61) and increased expression of NF-E2-related factor 2 (Nrf2) to protective from high glucose-induced oxidative stress, injury and inflammation in human renal tubular epithelial cells (62). For other CD38 inhibitors, 78c increased NAD^+^ to activate sirtuins, and AMPK, further improved glucose tolerance, muscle function, exercise capacity, and cardiac function in aged mouse model (63). Quercetin inhibited LPS-induced inflammation in macrophages via suppressing NF-κB signaling activation to relieve kidney inflammation and protect from AKI (64) (Figure 3B and Table 1).

## Conclusions

The regulation of intracellular NAD^+^ levels has become a crucial direction for exploring the potential mechanisms of multiple age-related metabolic diseases, including DKD. The regulation of NAD^+^ levels mainly involves two aspects, synthesis and catabolism. Many studies have shown that increasing the precursors and intermediates of NAD^+^ can benefit via increasing intracellular NAD^+^ levels, but some studies also have shown that exogenous supplementation or activation of some certain NAD^+^ synthesis key enzymes (such as NAMPT) may play a negative role in metabolic pathways. Further studies are still needed to confirm the therapeutic effects of supplementing precursors and intermediates on DKD. The results of studies on NAD^+^ catabolism are relatively certain. On the one hand, activation of the sirtuins family (especially SIRT1, 2, 3, 6) of NAD^+^-dependent deacetylases can inhibit mitochondrial oxidative stress, improve mitochondrial biogenesis, alleviate inflammation and reduce apoptosis, thereby preventing the progression of DKD. On the other hand, by inhibiting NAD^+^-consuming enzymes such as PARPs and cADPR synthetase (especially CD38), also ameliorate mitochondrial oxidative stress, inflammation and apoptosis in DKD. There have been a variety of drugs targeting the NAD^+^-consuming enzymes, such as activators of the sirtuins family and inhibitors of PARPs and cADPR, which is benefit for the treatment of DKD in cell and animal models. The regulation of NAD^+^ catabolism may be a potential target for the treatment of DKD.

## Author Contributions

JX contributed to drafting and writing the article. MK and DK contributed to the discussion of the review. MK is responsible for the integrity of the content. All authors revised the manuscript critically for important intellectual content and approved the final version to be published.

## Conflict of Interest

Boehringer Ingelheim, Mitsubishi Tanabe Pharma, Taisho Pharmaceutical Co. and Ono Pharmaceutical Co. contributed to establishing the Division of Anticipatory Molecular Food Science and Technology. The authors declare that the research was conducted in the absence of any commercial or financial relationships that could be construed as a potential conflict of interest.
